# Specificity of striatal dopamine D_1_ system in humans: implications for clinical use of D_1_ receptor-agonists in Parkinson's disease

**DOI:** 10.3389/fnhum.2023.1178616

**Published:** 2023-04-26

**Authors:** Satoshi Goto

**Affiliations:** ^1^Center for Drug Discovery and Development Sciences, Research Organization of Science and Technology, Ritsumeikan University, Kyoto, Japan; ^2^Department of Clinical Neuroscience, Institute of Biomedical Sciences, Tokushima University, Tokushima, Japan

**Keywords:** dopamine D_1_ receptors, dopamine D_1_ agonist, striatum, Parkinson's disease, basal gangalia, drug therapy, patient

## Introduction

Dopamine receptor-mediated signaling in the mammalian striatum, which comprises two functional subdivisions, i.e., the striosome and matrix compartments (Graybiel, 1990; Gerfen, 1992), serves as a principal determinant of basal ganglia function (Graybiel, 2008; Kreitzer, 2009; Gerfen and Surmeier, 2011). Its deregulation underlies pathophysiology and symptomatology of various basal ganglia disorders including Parkinson's disease (PD) (Crittenden and Graybiel, 2011). Striatal dopamine deficiency is the principal cause of motor symptoms in PD (Hornykiewicz, 2017). The administration of L-3,4-dihydroxyphenylalanine (L-DOPA), a dopamine prodrug that acts as the full agonist of both the dopamine D_1_- and D_2_-type receptors (D_1_Rs and D_2_Rs), is the most effective and commonly used for the treatment of PD (Hornykiewicz, 2017). However, long-term daily exposure to L-DOPA often causes troublesome adverse effects, such as L-DOPA-induced dyskinesia (LID) (Jenner, 2008; Calabresi et al., 2010; Bastide et al., 2015; Goto, 2017). Because of their longer half-lives and durations of action than those of L-DOPA, dopamine receptor agonists are currently used as an effective therapy for PD, although they primarily target D_2_Rs (Poewe et al., 2017). D_1_R-selective agonists (D_1_-agonists) have long been considered potential therapies for PD (for review see, Jones-Tabah et al., 2022). Like D_2_-agonists, D_1_-agonists improve motor deficits sufficiently in “rodent” models of PD; however, therapeutic trials with D_1_-agonists have failed to identify clinically applicable strategies because of their limited efficacy and the adverse effects induced by specific ligands (Jones-Tabah et al., 2022). Supplementary Table 1 shows the representatives of D_1_-agonists used in clinical trials for PD. Thus, there seems to be a species difference in the therapeutic efficacy of D_1_-agonists on motor symptoms under PD conditions. This “Opinion” article introduces anatomical evidence that, unlike in mice, a marked compartmental difference exists in the abundance of D_1_Rs in the human striatum, with a pronounced enrichment of D_1_Rs in the striosome compartment, but a relative paucity in the matrix compartment (Morigaki and Goto, 2015). The specificity of the striatal dopamine D_1_ system in humans is important when considering the therapeutic effects of D_1_-agonists in patients with PD.

## Striatal dopamine system in the functional anatomy of the basal ganglia

**Figure 1:**
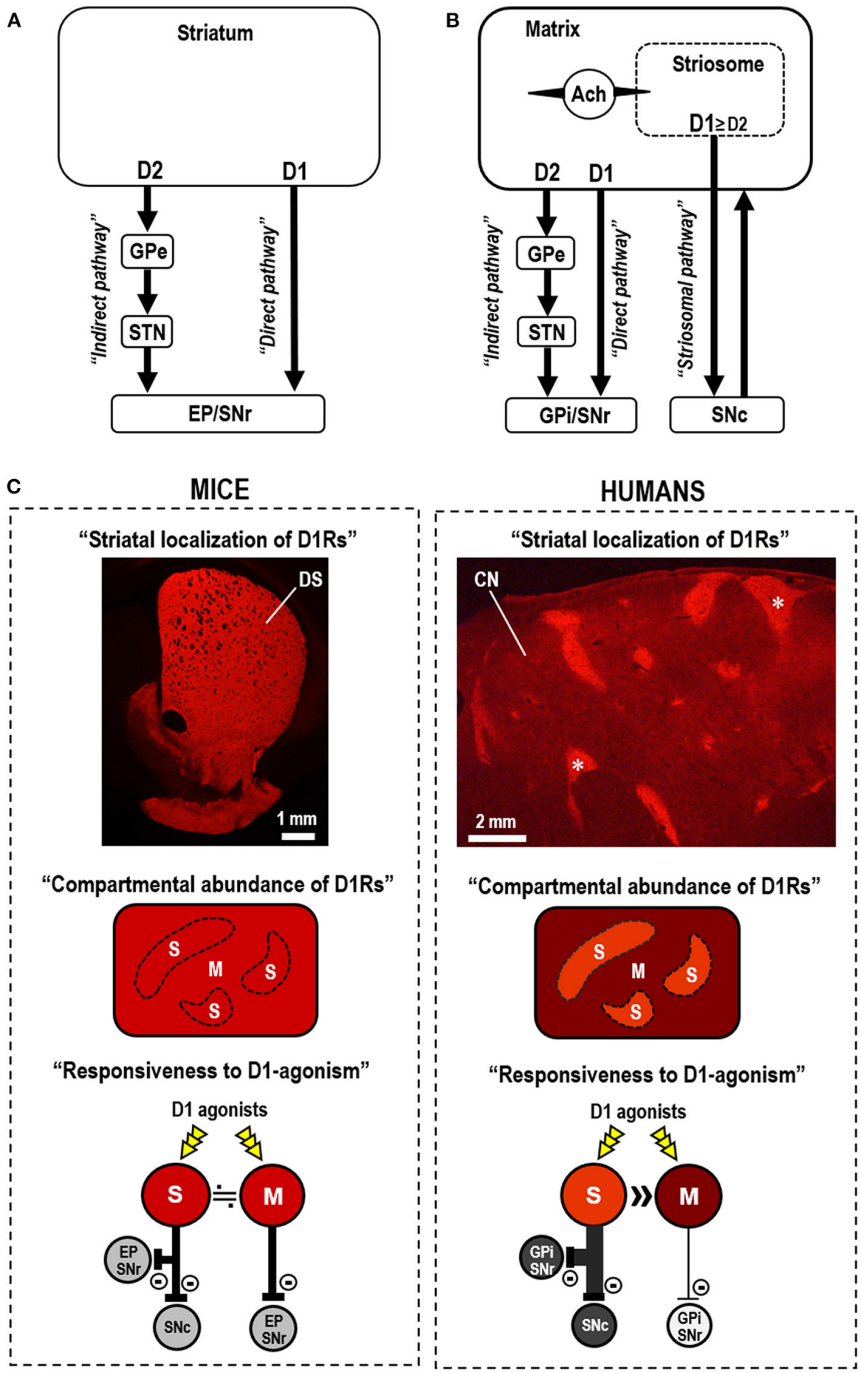
Striatal dopamine D_1_ systems in the functional anatomy of the basal ganglia. **(A)** Direct-indirect pathway model of the basal ganglia circuit (Alexander et al., 1986; Albin et al., 1989). The direct-indirect pathway model suggests that striatal dopamine D_1_ receptor (D_1_R)-expressing medium spiny neurons (MSNs) primarily form the direct pathway that targets the globus pallidus internus (GPi)/entopeduncular nucleus (EP) and substantia nigra pars reticulata (SNr). D_2_R-expressing MSNs form the indirect pathway that targets the globus pallidus externa (GPe), which sends projections to the subthalamic nucleus (STN). **(B)** In the three-pathway model of striatal efferent connectivity (Graybiel et al., 2000; Crittenden and Graybiel, 2011), striosomal MSNs form the striosome-specific pathway (striosomal pathway) that targets the substantia nigra pars compacta (SNc), which projects back to the entire striatum. Most MSNs that form the direct and indirect pathways lie in the matrix compartment. Striosomal MSNs also communicate with adjacent matrix MSNs via cholinergic interneurons (marked by Ach). The collaterals of the striosomal projections in GPi/EP and GPe are not shown in this diagram. **(C)** Compartmental differences in the abundance of D_1_Rs and responsiveness to D_1_R activation in mice (left) and humans (right). In mice, D_1_Rs are abundant in both the striosome (S) and matrix (M) compartments in the dorsal striatum (DS), with no apparent compartmental difference in abundance. This is evident in the immunofluorescence image of a mouse striatal section stained for D_1_Rs (Morigaki et al., 2017). Thus, no difference in the response to D_1_R-selective agonists (D_1_-agonists) is apparent between the striosome and matrix compartments in the mouse striatum. In humans, a marked compartmental difference exists in the abundance of D_1_Rs in the neostriatum. This is evident in the immunofluorescence image of a caudate nucleus (CN) section stained for D_1_Rs (Morigaki and Goto, 2015). Examples of the striosomes are indicated by asterisks. Thus, in humans, D_1_-agonists act preferentially in the striosomes that send the striosomal pathway, but not in the matrix compartment that forms the direct pathway.

Dopamine receptors, which belong to a superfamily of G-protein-coupled receptors (Missale et al., 1998), are categorized into two subclasses, D_1_Rs and D_2_Rs, respectively, based on their ability to elicit and inhibit the adenylyl cyclase-mediated production of 3',5'-cyclic adenosine monophosphate (cAMP) via the specific targeting of G-proteins (Kebabian and Calne, 1979; Missale et al., 1998). Striatal dopamine/cAMP signaling is integrated by medium spiny neurons (MSNs), which constitute more than 90% of the neuronal types in the striatum (Kreitzer, 2009; Crittenden and Graybiel, 2011; Gerfen and Surmeier, 2011; Goto, 2017). Striatal MSNs can be divided into two distinct subclasses based on their efferent projections, which form the “direct” striatonigral and “indirect” striatopallidal pathways, which mainly express D_1_Rs and D_2_Rs, respectively (Alexander et al., 1986; Albin et al., 1989). Both direct and indirect pathway MSNs form fundamental circuits in the basal ganglia, where D_1_Rs boost the excitability of striatonigral MSNs, whereas D_2_Rs diminish the excitability of striatopallidal MSNs. Thus, the “classical” direct-indirect pathway model (Figure 1A) suggests that the basal ganglia regulate the release and inhibition of movements via the D_1_-direct and D_2_-indirect pathways, respectively (Alexander et al., 1986; Albin et al., 1989).

The human neostriatum (known in rodents as the dorsal striatum) comprises the striosome (patch) and matrix compartments (Graybiel, 1990; Gerfen, 1992). Considering the striatal compartments, Graybiel et al. (2000) proposed a three-pathway model of striatal efferent connectivity (Figure 1B) (Graybiel et al., 2000; Crittenden and Graybiel, 2011), in which striosomal MSNs form a striosome-specific pathway targeting the substantia nigra pars compacta (SNc), with collaterals in the globus pallidus internus/entopeduncular nucleus and globus pallidus externus. Subsequently, most MSNs that form the direct and indirect pathways lie in the matrix compartment (Graybiel et al., 2000; Crittenden and Graybiel, 2011), which comprises more than 80% of the striatum volume (Johnston et al., 1990). The striosome compartment purportedly participates in the striatal dopamine signaling homeostasis through its projections to dopamine-producing cells in the SNc that project back to the entire striatum (Graybiel et al., 2000; Crittenden and Graybiel, 2011). The striosome compartment also regulates the activity of adjacent matrix MSNs via striatal interneurons (e.g., cholinergic interneurons) (Graybiel, 2008; Crittenden and Graybiel, 2011), which can regulate dopamine release (Threlfell and Cragg, 2011) and balance the activity between the direct and indirect pathways (Ding et al., 2011). Evidence also indicates that the striosome compartment mainly has afferent connections to limbic-related circuits, while the matrix compartment is related to associative and sensorimotor circuits (Gerfen, 1992; Graybiel, 2008; Crittenden and Graybiel, 2011). Therefore, the advanced model of basal ganglia circuits (Graybiel, 2008; Crittenden and Graybiel, 2011) suggests that the normal release of individual movements largely depends on the activity balance between the direct and indirect pathways arising from the matrix compartment, whereas the striosome compartment participates in the limbic control of motor behaviors.

## Specificity of the striatal dopamine D_1_ system in humans

As shown in the immunohistochemical studies on autopsied brains (Morigaki and Goto, 2015), D_1_Rs are differentially concentrated between the two striatal compartments in the human neostriatum (Figure 1C). Densitometric analysis reveals that the D_1_R density in striosomes is more than three times that in the matrix (Morigaki and Goto, 2015). However, no apparent compartmental difference in the abundance of D_1_Rs has been found in the dorsal striatum of mice (Figure 1C) (Morigaki et al., 2017). Since multiple neurochemical molecules immunohistochemically exhibit cross-species variations in their compartmental enrichments (Crittenden and Graybiel, 2011), the species difference in the compartmental abundance of D_1_Rs between humans and mice is not surprising and may parallel the phylogenic evolution of cortico-basal ganglia circuits (Hamasaki and Goto, 2019). However, the strategic localization of D_1_Rs in humans is crucial to the interdependent striatal dopamine signal processing of the respective compartments. This suggests that the striatal responsiveness to dopaminergic stimulation differs between the striosome and matrix compartments. As striatal MSNs are almost equally divided between D_1_R- and D_2_R-expressing MSNs (D_1_-MSNs and D_2_-MSNs) (Crittenden and Graybiel, 2011), the immunohistochemical results indicate that, in the human neostriatum, striosomal MSNs possess a greater abundance of D_1_R proteins than that in matrix MSNs. The specificity of the striosome-matrix dopamine system in humans is implicated in the clinical use of dopamine receptor agonists in the management of PD motor symptoms.

## Therapeutic effects of D_1_-agonists differ between mice and humans under PD conditions

Core symptoms of *de novo* patients with PD are characterized by a paucity of movement release during the execution of voluntary movements (Graybiel et al., 2000; Crittenden and Graybiel, 2011). Since the three-pathway model suggests that the normal release of individual movements depends on the activity balance between matrix-based direct and indirect pathways (Graybiel et al., 2000; Crittenden and Graybiel, 2011), striatal dopamine deficiency in the matrix compartment may represent the primary cause of these hypokinetic motor symptoms. As shown in Figure 1C, D_1_Rs are abundantly found in both the striosome and matrix compartments in the dorsal striatum of mice. However, the human neostriatum exhibits a pronounced enrichment of D_1_Rs in the striosomes, but a relative paucity of D_1_Rs in the matrix. This indicates that compared to striosomal D_1_-MSNs, D_1_-direct pathway MSNs in the matrix compartment respond poorly to D_1_-agonist exposure in humans. Therefore, the clinical use of D_1_-agonists has limited efficacy in treating hypokinetic motor symptoms in PD. Moreover, in humans, D_1_Rs are heavily enriched in the striosome compartment. This suggests that, when D_1_-agonists are systemically administered, they act preferentially in the striosome compartment, which purportedly participates in the limbic control of motor behaviors. Therefore, if a high dose of D_1_-agonists is administered to obtain high-yield therapeutic efficacy, it may simultaneously increase the potential risk of inducing LID, which is strongly linked to the over-activation of striosomal MSNs (Graybiel et al., 2000; Crittenden and Graybiel, 2011). This hypothesis conforms with evidence that D_1_-agonists tend to produce similar levels of dyskinesia as the D_1_/D_2_ agonist L-DOPA (Jones-Tabah et al., 2022). Moreover, the administration of D_1_-agonists may cause an undesired reduction in striatal dopamine content by activating the striosomal D_1_-MSNs that send inhibitory projections to the dopaminergic cells in the SNc (Graybiel et al., 2000; Crittenden and Graybiel, 2011). Therefore, a *single* use of D_1_-agonists may not represent an ideal therapy for PD in a clinical setting, although it may exert therapeutic effects on other clinical symptoms associated with PD (e.g., “off period” dystonia), possibly due to the loss of striosomal D_1_ signaling (Crittenden and Graybiel, 2011).

## Conclusion and future directions

The activity balance of D_1_R-mediated signaling between the striosome and matrix compartments serves as a key regulator of basal ganglia functions. An advanced model of the functional anatomy of the basal ganglia (Graybiel et al., 2000; Crittenden and Graybiel, 2011) suggests that striatal MSNs form three major efferent projection systems (Figure 1B), wherein the D_1_-direct and D_2_-indirect pathways mainly arise from matrix MSNs, whereas the striosome-specific pathway arises from striosomal MSNs. The immunohistochemical results from mouse and human brains (Morigaki and Goto, 2015; Morigaki et al., 2017) revealed a marked compartmental difference in D_1_R abundance in the human neostriatum, unlike in the dorsal striatum of mice (Figure 1C). This anatomical evidence updates our understanding of the functional anatomy of the basal ganglia and suggests that D_1_R-mediated signals are mainly processed through striosome-based circuits in humans. Recognizing the specificity of the striosome-matrix dopamine D_1_ system in humans contributes to our understanding of the symptoms and therapies for movement disorders of basal ganglia origin. The striatal compartment-specific responsiveness to D_1_R activation suggests that D_1_-agonists preferentially act on striosomal D_1_-MSNs that form the striosomal pathway, but not on matrix D_1_-MSNs that form the direct pathway. Hence, the author has a negative opinion on the clinical development of a *single* use of D_1_-agonists for the treatment of PD, in which hypokinetic motor symptoms primarily result from dopamine deficiency in the matrix compartment. However, if D_2_-agonists are concurrently administered, D_1_-agonists could be useful tool to treat hypokinetic motor symptoms in patients with PD, as in the D_1_/D_2_ agonist L-DOPA therapy. D_1_/D_2_ dopamine receptor synergism has been suggested to underlie the network-level changes in basal ganglia activation (Capper-Loup et al., 2002). On one hand, the author posits the use of D_1_-agonists as an effective therapy for the treatment of dystonias, because the loss of striosomal dopamine signaling is considered a potential cause of dystonia symptoms (Goto et al., 2005, 2013; Sato et al., 2008; Crittenden and Graybiel, 2011). Indeed, it was recently found that dual dopaminergic modulation, which induces an increase in striatal D_1_-signals, could exert a therapeutic effect on blepharospasm, a focal dystonia (Matsumoto et al., 2022). In this context, it is also noteworthy that dystonia symptoms (e.g., blepharospasm) frequently occur in patients with PD (Shetty et al., 2019). Additionally, D_1_-agonists may provide a useful tool for the treatment of cognitive and behavioral disorders that purportedly result from functional impairments of the associative and limbic brain circuits (Graybiel, 2008: Amemori et al., 2011; Crittenden and Graybiel, 2011). Understanding the specificity of the striosome-matrix dopamine D_1_ system in humans is necessary to comprehend the functional pathology of basal ganglia disorders. Therefore, the validity of the clinical development of dopaminergic modulators (e.g., D_1_-agonists) must be determined for the treatment of basal ganglia disorders including PD. Finally, the author hopes to develop *in vivo* brain imaging techniques that provide insight into the functions of the striosome-matrix dopamine D_1_ system in the human striatum.

## Author contributions

SG: conceptual design, execution, analysis, writing, and editing final version of the manuscript.
