# Supplementary material for: Specificity of striatal dopamine D1 system in humans: implications for clinical use of D1 receptor-agonists in Parkinson's disease
Source: Front Hum Neurosci. 2023 Apr 26;17:1178616. doi: 10.3389/fnhum.2023.1178616 (PMC10169585; doi:10.3389/fnhum.2023.1178616)
Supplement: Supplementary file 1 [file Table_1.DOCX]

**Supplementary Table 1.**　Representatives of dopamine D_1_ receptor (D_1_R)-selective agonists used in clinical trials for Parkinson’s disease (PD).

| Specific Ligands (chemical classes) | Affinity for D_1_Rs | Therapeutic applications in PD patients |
| --- | --- | --- |
| SKF 38393  (benzazepine agonist) | Partial D1/D5 agonist | No improvement of PD motor symptoms ([Braun](https://pubmed.ncbi.nlm.nih.gov/?term=Braun+A&cauthor_id=2949059) et al., 1987). |
| CY 208-243  (benzazepine agonist) | Partial D1/D5 agonist | Insufficient efficacy in treating PD motor symptoms (Tsui et al., 1989; Emre et al., 1992) |
| DAR-0100  (THIQ agonist) | Full or partial D1/D5 agonist | Promising anti-parkinsonian effects (Blanchet et al.,1998), but precluded due to poor pharmacokinetics and adverse effects (Salmi et al., 2004). |
| ABT-431  (THIQ agonist) | Full D1/D5 agonist | Promising anti-parkinsonian effects but poor oral bioavailability (Giardina and Williams, 2001; Rascol et al., 2001). |
| PF-06649751  (Non-catecholamine agonist) | Partial D1/D5 agonist | Promising anti-parkinsonian effects ([Gurrell](https://pubmed.ncbi.nlm.nih.gov/?term=Gurrell+R&cauthor_id=29478239) et al., 2018; Riesenberg et al., 2020), and the Phase III clinical trial is setting. |
| PF-06412562  (Non-catecholamine agonist) | Partial D1/D5 agonist | No further trials due to limited therapeutic efficacy (Papapetropoulos et al., 2018; Huang et al., 2020). |
| PF-06669571  (Non-catecholamine agonist) | Partial D1/D5 agonist | No further trials due to limited therapeutic efficacy (Gurrell et al., 2018). |

THIQ = tetrahydroisoquinoline, DAR-0100 = Dihydrexidine, PF-06649751 = Tavapandon.

**REFERENCES (for Supplementary Table 1)**

Blanchet PJ, Fang J, Gillespie M, [Sabounjian](https://pubmed.ncbi.nlm.nih.gov/?term=Sabounjian+L&cauthor_id=9844789) L, [Locke](https://pubmed.ncbi.nlm.nih.gov/?term=Locke+KW&cauthor_id=9844789) KW, [Gammans](https://pubmed.ncbi.nlm.nih.gov/?term=Gammans+R&cauthor_id=9844789) R, et al. Effects of the full dopamine D1 receptor agonist dihydrexidine in Parkinson’s disease. *Clin Neuropharmacol.* (1998) 21: 339–43.

[Braun](https://pubmed.ncbi.nlm.nih.gov/?term=Braun+A&cauthor_id=2949059) A, [Fabbrini](https://pubmed.ncbi.nlm.nih.gov/?term=Fabbrini+G&cauthor_id=2949059) G, [Mouradian](https://pubmed.ncbi.nlm.nih.gov/?term=Mouradian+MM&cauthor_id=2949059) MM, [Serrati](https://pubmed.ncbi.nlm.nih.gov/?term=Serrati+C&cauthor_id=2949059) C, [Barone](https://pubmed.ncbi.nlm.nih.gov/?term=Barone+P&cauthor_id=2949059) P, [Chase](https://pubmed.ncbi.nlm.nih.gov/?term=Chase+TN&cauthor_id=2949059) TN. Selective D-1 dopamine receptor agonist treatment of Parkinson's disease. *J Neural Transm.* (1987) 68: 41-50.

**doi: 10.1007/BF01244638**

[Emre](https://pubmed.ncbi.nlm.nih.gov/?term=Emre+M&cauthor_id=1535688) M, [Rinne](https://pubmed.ncbi.nlm.nih.gov/?term=Rinne+UK&cauthor_id=1535688) UK, [Rascol](https://pubmed.ncbi.nlm.nih.gov/?term=Rascol+A&cauthor_id=1535688) A, [Lees](https://pubmed.ncbi.nlm.nih.gov/?term=Lees+A&cauthor_id=1535688) A, [Agid](https://pubmed.ncbi.nlm.nih.gov/?term=Agid+Y&cauthor_id=1535688) Y, [Lataste](https://pubmed.ncbi.nlm.nih.gov/?term=Lataste+X&cauthor_id=1535688) X. Effects of a selective partial D1 agonist, CY 208-243, in de novo patients with Parkinson disease. *Mov Disord.* (1992) 7: 239-43.

**doi: 10.1002/mds.870070309**

Gurrell R, [Duvvuri](https://pubmed.ncbi.nlm.nih.gov/?term=Duvvuri+S&cauthor_id=29478239) S, [Sun](https://pubmed.ncbi.nlm.nih.gov/?term=Sun+P&cauthor_id=29478239) P, [DeMartinis](https://pubmed.ncbi.nlm.nih.gov/?term=DeMartinis+N&cauthor_id=29478239) N. A phase I study of the safety, tolerability, pharmacokinetics, and pharmacodynamics of the novel dopamine D1 receptor partial agonist, PF-06669571, in subjects with idiopathic Parkinson's disease. *Clin Drug Investig.*  (2018) 38: 509-17.

**doi: 10.1007/s40261-018-0632-6**

Huang X, Lewis MM, Van Scoy LJ, De Jesus S, Eslinger PJ, Arnold AC, et al. [The D1/D5 Dopamine partial agonist PF-06412562 in advanced-stage Parkinson's disease: a feasibilitysStudy.](https://pubmed.ncbi.nlm.nih.gov/32986682/) *J Parkinsons Dis.* (2020) 10:1515-27.

**doi: 10.3233/JPD-202188**

Papapetropoulos S, Liu W, Duvvuri S, Thayer K, Gray DL. Evaluation of D1/D5 partial agonist PF-06412562 in Parkinson's disease following oral administration. *Neurodegener Dis.* (2018) 18, 262–9.

**doi: 10.1159/000492498**

[Rascol](https://pubmed.ncbi.nlm.nih.gov/?term=Rascol+O&cauthor_id=11176963) O, [Nutt](https://pubmed.ncbi.nlm.nih.gov/?term=Nutt+JG&cauthor_id=11176963) JG, [Blin](https://pubmed.ncbi.nlm.nih.gov/?term=Blin+O&cauthor_id=11176963) O, [Goetz](https://pubmed.ncbi.nlm.nih.gov/?term=Goetz+CG&cauthor_id=11176963) CG, [Trugman](https://pubmed.ncbi.nlm.nih.gov/?term=Trugman+JM&cauthor_id=11176963) JM, [Soubrouillard](https://pubmed.ncbi.nlm.nih.gov/?term=Soubrouillard+C&cauthor_id=11176963) C, et al. Induction by dopamine D1 receptor agonist ABT-431 of dyskinesia similar to levodopa in patients with Parkinson disease. *Arch Neurol.* (2001) 58: 249-54.

**doi: 10.1001/archneur.58.2.249**

Riesenberg R, Werth J, Zhang Y, Duvvuri S, Gray D. PF-06649751 efficacy and safety in early Parkinson's disease: a randomized, placebo-controlled trial. *Ther Adv Neurol Disord.* (2020) 13:1756286420911296.

**doi: 10.1177/1756286420911296**

Salmi P, Isacson R, Kull B. (2004). Dihydrexidine–the first full dopamine D1 receptor agonist. *CNS Drug Rev.* (2004) 10: 230–42.

**doi: 10.1111/j.1527-3458.2004.tb00024.x**

[Tsui](https://pubmed.ncbi.nlm.nih.gov/?term=Tsui+JK&cauthor_id=2725884) JK, [Wolters](https://pubmed.ncbi.nlm.nih.gov/?term=Wolters+EC&cauthor_id=2725884) EC, [Peppard](https://pubmed.ncbi.nlm.nih.gov/?term=Peppard+RF&cauthor_id=2725884) RF, [Calne](https://pubmed.ncbi.nlm.nih.gov/?term=Calne+DB&cauthor_id=2725884) DB. A double-blind, placebo-controlled, dose-ranging study to investigate the safety and efficacy of CY 208-243 in patients with Parkinson's disease. *Neurology.*(1989) 39: 856-8.

**doi: 10.1212/wnl.39.6.856.**
